# The role of allogeneic hematopoietic cell transplantation for chronic lymphocytic leukemia: A review

**DOI:** 10.3389/fonc.2022.1105779

**Published:** 2023-01-18

**Authors:** Robert Puckrin, Mona Shafey, Jan Storek

**Affiliations:** Department of Hematology and Hematologic Malignancies, Tom Baker Cancer Centre and University of Calgary, Calgary, AB, Canada

**Keywords:** chronic lymphocytic leukemia (CLL), allogeneic hematopoietic cell transplant, graft-versus-hose disease (GvHD), Conditioning, graft-versus-leukemia (GvL)

## Abstract

Although the use of allogeneic hematopoietic cell transplantation (HCT) for chronic lymphocytic leukemia (CLL) has declined with the development of novel targeted agents, it continues to play an important role for eligible patients with high-risk or heavily pretreated CLL who lack other treatment options. CLL is susceptible to a potent graft-versus-leukemia (GVL) effect which produces long-lasting remissions in 30-50% of transplanted patients. While allogeneic HCT is associated with significant risks of graft-versus-host disease (GVHD), infection, and non-relapse mortality (NRM), improvements in patient and donor selection, reduced intensity conditioning (RIC), GVHD prophylaxis, and supportive care have rendered this an increasingly safe and effective procedure in the current era. In this review, we discuss recent advances in allogeneic HCT for CLL, with a focus on the optimal evidence-based strategies to maximize benefit and minimize toxicity of this potentially curative cellular therapy.

## Introduction

The advent of novel therapies has revolutionized the treatment of CLL, leading to a persistent decline in the use of allogeneic HCT for this disease (1, 2). However, the powerful GVL effect of allogeneic HCT offers the potential for long-term remissions in an otherwise incurable malignancy. Allogeneic HCT still plays an important role in the treatment of CLL, particularly for eligible patients with high-risk genetic features and those with resistance to Bruton tyrosine kinase (BTK) and/or B-cell lymphoma-2 (BCL2) inhibitors who have limited other therapeutic options (3). In this review, we describe recent advances in allogeneic HCT and the optimal approaches to the application of this cellular therapy in CLL (**Table 1**).

**Table 1:** Evidence-based practice recommendations for allogeneic HCT in CLL.

• Allogeneic HCT should be reserved for medically fit patients with high-risk CLL who lack other treatment options, such as those with heavily pre-treated CLL, high-risk genetic features, and/or resistance to BTK and/or BCL2 inhibitors • The timing of allogeneic HCT must be individualized to each patient but should ideally be performed while CLL remains well controlled • A fully-HLA matched related or unrelated donor is generally preferred, but there is accumulating evidence that haploidentical transplantation results in favorable outcomes with PTCy-based GVHD prophylaxis • Given the familial predisposition of CLL, screening peripheral blood flow cytometry should be considered to rule out MBL or CLL in related stem cell donors • NMA or RIC is generally preferred over MAC to reduce the risk of GVHD and NRM after allogeneic HCT in CLL • Calcineurin inhibitors in combination with an antimetabolite are the most frequently used GVHD prophylaxis in CLL • Randomized trials have shown benefit with *in-vivo* T-cell depletion using ATG or PTCy in various hematologic malignancies, although further research is needed to confirm their impact on GVHD and GVL in CLL • Treatment of relapsed CLL after allogeneic HCT may include withdrawal of immunosuppression, donor lymphocyte infusions, BTK or BCL2 inhibitors, or enrolment in a clinical trial • Allogeneic HCT should also be considered for Richter transformation with high-risk features such as TP53 mutations or previously treated or clonally-related CLL

## Indications and timing of allogeneic HCT for CLL

CLL is diagnosed at the median age of 70 years and typically carries a favorable prognosis with a 5-year overall survival (OS) rate of 88% (4). Most patients can be successfully managed for many years with active surveillance or low-intensity approaches such as BTK and BCL2 inhibitors alone or in combination with anti-CD20 monoclonal antibodies or alternatively with conventional chemoimmunotherapy (5–9). As a result, most patients with CLL will never require allogeneic HCT during the course of their disease. However, there are important subsets of patients with CLL in whom allogeneic HCT is still warranted, including younger patients who eventually exhaust all available lines of therapy or those with poor prognosis disease characteristics, such as deletion 17p, TP53 aberrations, or complex karyotype (10, 11). Due to the risks of transplant-related morbidity and mortality (TRM), allogeneic HCT is usually reserved for medically fit patients who have limited other treatment options.

Although the benefits of allogeneic HCT in CLL have never been confirmed with a randomized controlled trial, large datasets demonstrate that allogeneic HCT achieves long-lasting remissions in up to 30-50% of patients with heavily pre-treated CLL, which is higher than would be expected with conventional therapies (12–14). Retrospective comparative studies have also suggested that allogeneic HCT is associated with a lower risk of relapse and improved survival compared to non-transplant approaches (15–18). These studies were largely conducted in the pre-BTK and BCL2 inhibitor era and must be interpreted cautiously due to the risk of selection bias, but they do provide some rationale to support the use of allogeneic HCT in high-risk CLL.

The indications for allogeneic HCT have evolved over the years in accordance with the rapidly changing treatment landscape of CLL (2, 5, 19–21). Factors to be taken into consideration when assessing eligibility for allogeneic HCT include patient values, age, performance status, comorbidities, donor availability, deletion 17p and TP53 status, prior treatment history and duration of response, depth of response to current therapy, and alternative treatment options. The 2016 American Society for Transplantation and Cellular Therapy (ASTCT) guidelines recommend allogeneic HCT for eligible patients with relapsed standard-risk CLL who develop BTK inhibitor resistance, and for high-risk patients with deletion 17p, TP53 aberrations, and/or complex karyotype who have relapsed after 2 lines of therapy and/or a BTK or BCL2 inhibitor (2). The 2018 European Society for Blood and Marrow Transplantation (EBMT) guidelines state that allogeneic HCT should be recommended for eligible patients responding to a second targeted agent following resistance to both chemoimmunotherapy and a first targeted agent, and that it may be considered for select patients at low risk of TRM who have TP53 aberrations but are responding to a first targeted agent after previous chemoimmunotherapy failure (19). The current National Comprehensive Cancer Network (NCCN) guidelines recommend allogeneic HCT solely for fit patients with relapsed/refractory CLL after prior treatment with both a BTK and BCL2 inhibitor (22). These patients with “double refractory” CLL have a poor prognosis with median survival of 3.6 months, representing a critical area of unmet need in whom allogeneic HCT can be considered (3, 23). Finally, allogeneic HCT may also be warranted for patients with Richter transformation, as discussed later in this article.

While the optimal timing of allogeneic HCT must be individualized to each patient, it should ideally be performed while the CLL remains well controlled, given that disease status is an important predictor of relapse risk and GVHD (24). For patients with heavily pretreated CLL who prioritize the chance of long-term disease control over potential transplant-related toxicities, it may thus be preferable to proceed to allogeneic HCT while still responding to a second targeted agent, which is usually a BTK or BCL2 inhibitor (19). The median progression-free survival (PFS) of venetoclax is as short as 24 months for patients previously exposed to a BTK inhibitor (25), so it may be reasonable to proceed to allogeneic HCT within the first 1-2 years of commencing venetoclax to avoid risking the loss of remission. A similar timeline can be considered for patients with venetoclax resistance who are responding to a BTK inhibitor, which has a median PFS of 32-34 months in this setting (26, 27). Of note, these data are largely derived from patients receiving targeted agents after prior chemoimmunotherapy, and the timing of transplantation is less clear in the current era of upfront treatment with BTK or BCL inhibitors. It is the opinion of the authors that allogeneic HCT may be reasonably considered for patients with refractory disease and/or short-lived responses to both targeted agents if no clinical trials or other novel therapies are available, given the low probability of achieving a durable response to chemoimmunotherapy following BTK and BCL2 inhibitor failure. In such cases, there is limited data to guide the selection of pre-transplantation bridging therapies for patients who have already exhausted both BTK and BCL2 inhibitors (28). Alternative agents may be used to induce a response prior to allogeneic HCT, including non-covalent BTK inhibitors (29), PI3K inhibitors (30), alemtuzumab (31), anti-CD20 monoclonal antibodies (32), chemoimmunotherapy (33), rechallenging with a BTK inhibitor or venetoclax (27, 34, 35), or enrolment in a clinical trial.

## Graft-versus-leukemia effect in CLL

The curative potential of allogeneic HCT in CLL arises from the ability of donor immune effector cells to recognize and eradicate recipient leukemia cells. Evidence to support a strong GVL effect in CLL includes the following: long-term remission of CLL can be achieved with low-intensity non-myeloablative (NMA) conditioning and is more likely to occur for CLL than other hematologic malignancies (14, 36, 37); rapid or complete donor T-cell engraftment is associated with faster and more durable tumor clearance (38–41); the development of chronic GVHD is protective against relapse (42, 43); and responses can be induced by withdrawal of immunosuppression and/or donor lymphocyte infusion (DLI) (41, 44, 45). Of note, the GVL effect is frequently a delayed process in CLL, with a gradual reduction in tumor burden occurring over a period of up to 6-11 months after allogeneic HCT (44, 46)

## Donor selection and stem cell source

As with other diseases, HLA matching is an important determinant of outcomes of allogeneic HCT for CLL (47), and a fully HLA-matched related donor (MRD) or unrelated donor (MUD) is generally preferred although a haploidentical related donor is also acceptable (48–50). Data from the Center for International Blood and Marrow Transplant Research (CIBMTR) show that among 1,782 allogeneic HCT recipients with CLL, 3-year OS was 62% with a MRD, 53% with a MUD, 58% with a haploidentical donor, and 49% with a mismatched unrelated donor (MMUD) (50). In addition to HLA matching, other prognostic factors to be considered during donor selection include prioritization of younger donor age (51), preference for cytomegalovirus (CMV) matching (52), and selection of a male donor for a male recipient (53, 54).

The use of a haploidentical donor with post-transplant cyclophosphamide (PTCy)-based GVHD prophylaxis appears to result in lower risks of GVHD and comparable survival outcomes as a MRD or MUD in other lymphoid and myeloid malignancies (55, 56). In CLL, the Baltimore group reported 4-year OS 52% and progression-free survival (PFS) 37% among 64 patients undergoing NMA haploidentical HCT with PTCy prophylaxis, with grade II-IV acute GVHD incidence of 27% and chronic GVHD incidence of 17% (57). An EBMT study of 117 patients who underwent haploidentical HCT for CLL (of whom only 38% received PTCy) reported 2-year OS 48% and PFS 38%, with grade II-IV acute GVHD 32% and chronic GVHD not reported (58).

Umbilical cord blood (UCB) transplantation is performed less frequently in the adult population due to concerns regarding low CD34+ stem cell dose, delayed engraftment, and heightened risks of infection and NRM (59). However, a study of 68 patients with CLL who underwent UCB transplantation reported 3-year OS 54% and PFS 45%, suggesting this may be a viable option for individuals lacking other suitable donors (60). Another emerging alternative is MMUD HCT with PTCy prophylaxis, with a phase II trial including 3 patients with CLL showing encouraging survival outcomes even among recipients with donors matched at 4-6 of 8 HLA alleles (61).

A question unique to CLL is whether related stem cell donors should undergo screening for CLL and its precursor condition monoclonal B-cell lymphocytosis (MBL). CLL has a strong familial predisposition, and CLL or MBL clones can be identified in up to 13-16% of first-degree relatives of patients with CLL, with increasing incidence with age (62–64). Multiple case reports have documented the transmission of CLL from donor to recipient *via* allogeneic HCT (65–70). As such, the ASTCT recommends that potential related donors undergo screening with peripheral blood flow cytometry to reduce the risk of transmission of a malignant or premalignant clone to the recipient (2).

The optimal stem cell source for allogeneic HCT is a matter of debate, and there have been no large studies addressing this question in CLL. Multiple randomized trials comparing marrow to peripheral blood stem cells (PBSC) for hematologic malignancies have been conducted (71, 72). A meta-analysis of these studies showed a lower risk of grade 3-4 acute and extensive chronic GVHD but a higher risk of relapse with marrow, with similar OS except in a subgroup of patients with high-risk disease (72). Although the reduced risk of GVHD could potentially make marrow an attractive option for CLL, the lack of survival advantage in most patients and the logistic simplicity of PBSC collection has rendered it the preferred stem cell source used in >90% of allogeneic HCT for CLL (2, 12, 24).

## Conditioning regimens

Pre-transplant conditioning plays an important role in cytoreduction and contributes to long-term disease control after allogeneic HCT independently of the GVL effect (66). In a randomized trial enrolling younger patients (age <65 years) with acute myeloid leukemia (AML) or myelodysplastic syndrome (MDS), myeloablative conditioning (MAC) was shown to confer a survival advantage over RIC, with the significantly lower relapse risk of MAC offsetting its higher risks of acute and chronic GVHD and NRM (73, 74). However, it is unknown if these results can be extrapolated to CLL, given the generally indolent disease course, older and more comorbid patient population, increased risks of infection from underlying CLL-related immune dysregulation, and potentially greater chemoresistance induced by multiple previous lines of therapy. Retrospective studies have reported that MAC is associated with prohibitively high rates of NRM at 30-50% in CLL, whereas RIC is associated with lower NRM and potentially improved overall survival (13, 24, 75–78). In addition, a systematic review and meta-analysis of 48 studies including 1,903 patients with CLL concluded that RIC regimens are associated with lower NRM and slightly improved OS compared to MAC (79). Of note, these retrospective analyses must be interpreted with caution due to the paucity of randomized data and inherent risk of selection bias. Nevertheless, MAC has largely fallen out of favor for CLL and the majority (>75-80%) of allogeneic HCT performed for this indication employ RIC (12, 80). ASTCT guidelines recommend RIC or NMA for all patients with CLL undergoing allogeneic HCT (2).

The optimal RIC protocol for CLL has not been determined. Drugs with known activity in CLL such as fludarabine are often incorporated (81–83), and the most commonly-used RIC regimens are fludarabine-busulfan 6.4mg/kg (Flu-Bu2) and fludarabine-melphalan (Flu-Mel) (80). Bendamustine-based conditioning has also shown promising results in small prospective trials (84). In addition, the inclusion of anti-CD20 monoclonal antibodies in conditioning may reduce the risk of CLL relapse after allogeneic HCT, although concerns of increased infection and NRM have been raised (85, 86). While comparative data of RIC regimens is lacking in CLL, a CIBMTR analysis of 1,823 patients undergoing RIC allogeneic HCT for non-Hodgkin lymphoma (NHL) found that Flu-Bu2 and fludarabine-cyclophosphamide (Flu-Cy) were associated with lower NRM and favorable OS compared to Flu-Mel140mg/m^2^ (87). There is also growing interest in the use of treosulfan as a low-toxicity alternative to busulfan in RIC regimens, and a randomized trial in AML/MDS found that fludarabine-treosulfan (Flu-Treo) was associated with improved NRM and OS compared to Flu-Bu2 (88, 89). Preliminary data suggest that Flu-Treo may also be a viable conditioning regimen in NHL (90, 91), but confirmation in larger studies is required.

Given that CLL is particularly susceptibility to the GVL effect, NMA regimens have also been explored to harness the benefits of GVL while minimizing reliance on the cytoreductive but potentially toxic effects of conditioning (37). In a study of 82 patients with fludarabine-refractory CLL, NMA conditioning with 2 Gy TBI ± fludarabine achieved a complete response in 55% with 5-year OS 50%, PFS 39%, and NRM 23%, although high relapse rates were observed among patients with lymphadenopathy ≥5cm (36). Real-world outcomes of NMA and RIC appeared to be generally comparable in an EBMT study of 432 patients with CLL, with a trend to a lower risk of 100-day NRM with NMA at 3% versus 10% (14). NMA regimens can thus be considered a minimal toxicity alternative to RIC and may be favored for older, comorbid, or heavily pretreated patients with low tumor burden CLL.

Total body irradiation (TBI) is often incorporated into conditioning protocols to improve disease eradication, immune clearance, and donor engraftment. However, a CIBMTR analysis study of 897 patients with CLL and other lymphomas reported that the inclusion of low-dose (≤2 Gray [Gy]) TBI in NMA conditioning regimens does not appear to impact OS or PFS (92). Regarding TBI-based versus chemotherapy-only MAC, a CIBMTR study of 180 patients with CLL did not find a significant survival advantage, although there was a trend toward superior OS with TBI-based conditioning (93). As such, any potential role for TBI in allogeneic HCT for CLL is unclear.

## GVHD prophylaxis strategies

The cornerstone of GVHD prophylaxis in MRD or MUD HCT is a calcineurin inhibitor (CNI) combined with an antimetabolite such as methotrexate (MTX) or mycophenolate mofetil (MMF) (94). Small numbers of patients with CLL were enrolled in randomized trials demonstrating the efficacy of these combinations (95–97), and they are the most frequently employed GVHD prophylaxis strategies used for CLL in the real-world setting (13, 24). Sirolimus may be an acceptable alternative to methotrexate which is associated with similar risks of GVHD but faster engraftment and less mucositis (98). In addition, a phase III trial including 14 patients with CLL found that the addition of sirolimus to cyclosporine and MMF led to lower risks of acute GVHD and improved NRM and OS following NMA HCT (99).

T-cell depletion is another important prophylaxis strategy which targets the alloreactive donor T-cells believed to be responsible for GVHD. However, T-cell depletion is uncommonly utilized in allogeneic HCT for CLL, likely owing to concerns of potential disease relapse arising from an attenuated GVL effect (1, 13, 14, 100). Ex-vivo T-cell depletion (e.g., with CD34+ selection) is associated with excess risks of infection, NRM, and possibly relapse (101). Conversely, *in-vivo* T-cell depletion with ATG-Thymoglobulin^®^ has been shown in randomized trials to reduce the risks of acute and chronic GVHD after myeloablative HCT without worsening NRM or relapse (102–107). ATG was also associated with an OS advantage in one randomized study which included 16 patients with CLL and 64 recipients of RIC/NMA regimens (107). Thus, albeit promising, larger studies are needed to definitively confirm a role for ATG in allogeneic HCT for CLL. The anti-CD52 monoclonal antibody alemtuzumab is another intuitively attractive option for *in-vivo* T-cell depletion in CLL, given its activity as both a therapeutic for CLL and a GVHD prophylaxis agent (108–113). However, alemtuzumab has been linked to increased risks of relapse after allogeneic HCT for CLL (14, 47, 53, 114).

As alluded to earlier, it is anticipated that PTCy-based prophylaxis will play an increasing role in MRD and MUD HCT in the future, given its remarkable ability to overcome the HLA disparity of haploidentical and MMUD transplants (61, 101, 115, 116). In a randomized trial including 10 patients with CLL, the addition of PTCy to CNI plus MMF prophylaxis resulted in significant reductions in acute and chronic GVHD following MRD or MUD NMA HCT, without significantly impacting the risk of relapse or NRM (116). Similarly, the preliminary results from another randomized trial showed that the addition of PTCy to tacrolimus and MMF prophylaxis was associated with lower risks of acute and chronic GVHD in the setting of RIC HCT (117, 118). In light of these promising findings, further research is needed in larger cohorts of patients with CLL to confirm if ATG, PTCy, a combination of ATG and PTCy, or other emerging GVHD prophylaxis agents such as abatacept may improve the outcomes of allogeneic HCT for CLL by providing protection against GVHD while still preserving the GVL effect (119).

## Outcomes of allogeneic HCT for CLL

The outcomes of allogeneic HCT for CLL are heterogenous and vary in accordance with patient factors, transplant protocols, and time period (**Table 2**). In the largest study published to date of 2,589 patients with CLL who underwent allogeneic HCT between 2000-2010, the EBMT reported 5-year OS 45%, PFS 35%, NRM 36%, and relapse 29% (12). Similar findings were observed in a CIBMTR study of 606 patients with CLL who received RIC allogeneic HCT between 2008 and 2014, with 4-year OS 56% and PFS 41% (80). A systemic review and meta-analysis including 1,903 patients with CLL reported pooled rates of OS at 51-60%, PFS at 41-46%, and NRM at 23-32% after allogeneic HCT (79). Outcomes appear to have improved in the current era, possibly thanks to advances in patient/donor selection, conditioning protocols, supportive care, and the introduction of novel CLL therapies (121). As an example, in a report of 108 patients with CLL undergoing allogeneic HCT since 2010, 3-year OS was 73% and PFS was 61%, with cumulative incidences of NRM 14% and relapse 24% (100).

**Table 2:** Outcomes of allogeneic HCT for CLL from representative studies.

Study	Population	PFS	OS	Relapse	NRM	GVHD
Systematic review (79)	1903 recipients of MAC or RIC HCT	46% RIC vs 41% MAC	60% RIC vs 51% MAC	N/A	23% RIC vs 32% MAC	Grade 2-4 acute GVHD: 46% RIC vs 46% MAC Chronic GVHD: 55% RIC vs 59% MAC
EBMT registry study (14)	432 recipients of RIC or NMA HCT, 2000-2011	5-yr: 38% RIC vs 43% NMA	5-yr: 46% RIC vs 52% NMA	5-yr: 28% RIC vs 25% NMA	5-yr: 35% RIC vs 32% NMA	N/A
EBMT registry study (12)	2589 HCT recipients, 2000-2010	5-yr: 35% 10-yr: 28%	5-yr: 45% 10-yr: 35%	5-yr: 29% 10-yr: 32%	5-yr: 36% 10-yr: 40%	N/A
CIBMTR registry study (13)	297 recipients of MAC or RIC HCT, 1995-2007	5-yr: RIC 30% vs MAC 22%	5-yr: RIC 48% vs MAC 36%	5-yr: RIC 35% vs MAC 17%	5-yr: RIC 40% vs MAC 54%	Grade 2-4 acute GVHD: 35% RIC vs 49% MAC Chronic GVHD: 39% RIC vs 44% MAC
CIBMTR registry study (80)	606 recipients of RIC HCT, 2008-2014	4-yr 41%	4-yr 56%	N/A	N/A	N/A
Multicenter retrospective study (120)	65 HCT recipients after novel agent	2-yr 63%	2-yr 81%	2-yr 27%	2-yr 13%	Grade 2-4 acute GVHD: 37% Moderate/severe chronic GVHD: 27%
Single center retrospective study (100)	30 recipients of RIC HCT after novel agent	3-yr PFS 72%	3-yr OS 87%	3-yr 21%	3-yr 7%	Grade 3-4 acute GVHD: 13% Chronic GVHD: 57%

Long-term follow-up is essential to evaluating the curative potential of allogeneic HCT and the impact of late toxicities. In the 10-year follow-up of the CLL3X trial, long-lasting remissions were observed in approximately one-third of patients after RIC allogeneic HCT, with 10-year OS 51%, PFS 34%, relapse 46%, and NRM 20% (42). The EBMT also reported 10-year OS 35%, PFS 28%, relapse 32%, and NRM 40% after allogeneic HCT, with patients reaching the 5-year landmark having a 79% probability of remaining alive and in remission 10 years after transplant (12). It should be noted that the above studies did not have a control group and it is unclear whether the outcomes of allogeneic HCT are better than that of other modalities in the current era. Nevertheless, there appears to be a sizeable minority of allogeneic HCT recipients who are potentially cured of their CLL. Ongoing disease surveillance is still warranted as late relapses have been reported to occur more than 20 years after HCT (122). In addition, long-term survivors remain at persistently elevated risks of late NRM compared to the general population, highlighting the importance of comprehensive survivorship care after allogeneic HCT (12, 123).

While the use of allogeneic HCT has declined with the development of novel targeted agents for CLL, recent publications show it remains a reasonable option in the current era (30, 34, 100, 120). In one report of 30 patients with high-risk CLL previously treated with a BTK and/or BCL2 inhibitor, outcomes were excellent after RIC allogeneic HCT with 3-year OS 87% and PFS 72% (100). A separate study of 65 patients with high-risk, heavily pretreated CLL found similarly favorable outcomes with 2-year OS 81% and PFS 63% following allogeneic HCT after a previous BTK and/or BCL2 inhibitor (120). OS and PFS appeared similar regardless of whether patients had received one versus two or more novel agents or a BTK versus a BCL2 inhibitor as the most recent line of therapy before allogeneic HCT. Encouragingly, both studies also reported low risks of TRM at 7% and 13% with modern transplantation techniques (100, 120). It is conceivable that modern CLL therapies have the potential to improve the outcomes of allogeneic HCT by optimizing pre-transplant disease control or by inducing less toxicity compared to conventional chemotherapy.

Allogeneic HCT also has the potential to overcome the adverse prognosis associated with high-risk tumor biology in CLL (124, 125). While there is conflicting evidence that patients with deletion 17p and complex karyotype may have inferior PFS after allogeneic HCT (54, 80, 126–128), durable remissions are still achieved in a subset of patients with high-risk cytogenetics with reported OS 44-60% and PFS 29-43% (43, 129, 130). Other studies have reported that TP53 mutations may not significantly influence the outcomes of allogeneic HCT, although confirmation in larger datasets is needed (120, 131).

Disease status is another key determinant of outcomes after allogeneic HCT, with multiple studies consistently reporting lower relapse rates and improved PFS among patients in complete or partial response prior to transplantation (13, 14, 46, 128, 132). Nevertheless, up to 30-40% of patients with persistent or progressive CLL at the time of transplantation can still achieve long-term disease control (14, 30). Other factors associated with improved outcomes of allogeneic HCT in CLL include younger patient age, better performance status, lower HCT Comorbidity Index, fewer prior lines of therapy, better HLA matching, donor-recipient sex match, RIC rather than MAC, complete donor engraftment, and higher volume transplant centers (13, 39, 47, 53, 54, 100, 120, 128, 132, 133). Many of these factors have been incorporated into prognostic models proposed to predict survival outcomes after allogeneic HCT for CLL (43, 54, 80).

## Complications of allogeneic HCT

GVHD remains a significant complication of allogeneic HCT, and patients with CLL are at particularly elevated risks of both acute and chronic GVHD. In a systematic review of 48 studies evaluating allogeneic HCT for CLL, the pooled rates of grade 2-4 acute GVHD was 46% and chronic GVHD was 55-59% (79). Other studies have reported similar risks of grade 2-4 acute GVHD in 35-49% and chronic GVHD in 27-48% of patients (13, 100, 120). The most well-identified risk factor for acute and chronic GVHD in CLL is the use of MAC as opposed to RIC/NMA (13, 24, 43). Stable or progressive disease at the time of transplantation has also been associated with increased risks of acute GVHD, while poor performance status has been correlated with increased risks of chronic GVHD (13, 24). As with other malignancies, GVHD often represents a dual-edged sword in CLL. On one hand, GVHD is a leading cause of morbidity and mortality after allogeneic HCT (12, 13), while on the other the development of chronic GVHD has been shown to confer protection against relapse in CLL, likely owing to a stronger GVL effect (42, 43). As an example, in one study the risk of relapse was significantly lower among patients with versus without chronic GVHD at 19% versus 53% (43). Nevertheless, appropriate GVHD prophylaxis, monitoring, and treatment is needed to reduce the impact of this complication on the survival and quality of life of patients with CLL (134).

Patients with CLL are predisposed to infection due to humoral and cellular immune defects induced by underlying immune dysregulation and the effects of prior therapies (135), a problem further brought to light by the COVID-19 pandemic (136–138). They are thus particularly vulnerable to the severe immunosuppression induced by allogeneic HCT, and careful monitoring and prevention of infection with prophylactic antimicrobials and revaccinations is essential (139). Although the routine use of intravenous immunoglobulin (IVIg) replacement does not improve survival after allogeneic HCT (140), it can be considered to reduce the risk of infection among patients with CLL who have hypogammaglobulinemia and severe or recurrent infections (141). Patients with CLL are also at heightened risk of secondary malignancies after allogeneic HCT (142, 143), and regular cancer screening including annual skin examination is a critical piece of survivorship care (144).

## Monitoring and management of disease relapse after allogeneic HCT

Despite the potent GVL effect in CLL, relapse will ultimately occur in >30% of patients following allogeneic HCT (12). ASTCT guidelines recommend monitoring for measurable residual disease (MRD) with flow cytometry to track disease activity after allogeneic HCT (2). Varying patterns of post-transplant MRD kinetics have been identified which correlate with GVL activity (44, 45, 145), and the absence of MRD 1 year post-transplant is associated with a high likelihood of durable remission (145–148). In addition to its prognostic value, MRD monitoring may enable pre-emptive therapeutic intervention to reduce the risk of relapse after allogeneic HCT (148). In one prospective study, the administration of MRD-guided pre-emptive DLI resulted in achievement of MRD-negativity in 3 of 6 patients (114). This finding prompted a phase II multicenter trial of 42 patients with CLL who received risk-adapted immune intervention with immunosuppression withdrawal +/- DLI in response to serial MRD monitoring with peripheral blood flow cytometry after allogeneic HCT (45). This strategy appeared feasible and effective with 64% of patients achieving MRD negativity at 12 months, yielding a 3-year OS rate of 87% and PFS 63%. Further studies are needed to identify the optimal timepoints for MRD assessment and to confirm the clinical utility of pre-emptive immune intervention in CLL.

Whereas the relapse of acute leukemia after allogeneic HCT is typically associated with a poor prognosis (149), patients with CLL relapsing post-transplant may in fact experience relatively favorable outcomes owing to the availability of multiple rescue therapies (148, 150). In one study of 52 patients with CLL relapsing after allogeneic HCT, the overall response rate to subsequent post-transplant treatment was 45% and 2- and 5-year OS rates were 67% and 38%, respectively (150). Limited data suggest that withdrawal of immunosuppression and/or DLI can induce a GVL effect and achieve complete responses in 30-55% of patients relapsing after allogeneic HCT (41, 151, 152). The addition of rituximab to augment the effects of DLI has been evaluated in two studies with conflicting results; in one report, 20/43 (47%) patients achieved complete responses which tended to be durable, whereas in the second study a sustained remission was achieved in only 2/13 (15%) patients (148, 153). The potential benefit of DLI must also be weighed against the substantial risks of inducing clinically significant GVHD in up to 38-48% of patients (148, 153, 154). BTK and BCL2 inhibitors represent other highly effective treatment options for patients relapsing after allogeneic HCT who have not previously developed resistance to these therapies (155–158). Ibrutinib is a particularly attractive option in the post-transplant setting given its potential role in the treatment of chronic GVHD (159, 160). Finally, patients developing relapse after allogeneic HCT should be strongly considered for enrolment in clinical trials evaluating promising new therapies such as non-covalent BTK inhibitors or CAR-T cells (29, 161).

## Allogeneic HCT for histologic transformation of CLL

Richter transformation of CLL to an aggressive diffuse large B-cell lymphoma (DLBCL) occurs in >2-3% of patients and frequently portends a poor prognosis with median survival 3-11 months (162–164). Of note, a subset of patients with Richter transformation may in fact have relatively favorable outcomes akin to *de novo* DLBCL, particularly if they have previously untreated CLL, TP53 intact and clonally-unrelated Richter transformation, and demonstrate a complete response to R-CHOP (165–167). However, the majority of cases with heavily pre-treated CLL, TP53 mutations, or clonally related Richter transformation are at high risk of treatment failure, with PFS rates <25% after CHOP-based chemoimmunotherapy (168, 169). As a result, allogeneic HCT is recommended for eligible patients with high-risk Richter transformation responding to induction therapy (2, 170). In the largest study of 118 patients undergoing allogeneic HCT for Richter transformation, the CIBMTR reported 4-year OS 52%, PFS 43%, relapse incidence 30%, and NRM 27%. Superior outcomes were noted among patients in complete or partial response at the time of transplant, while the presence of deletion 17p, conditioning intensity, and previous treatment with BTK or BCL2 inhibitors did not have prognostic impact (171). The favorable outcomes of this study and others confirm that allogeneic HCT is the preferred consolidation strategy for eligible patients with high-risk Richter transformation (132, 172, 173). Alternatively, autologous HCT may be considered for selected patients with chemosensitive disease (171), while novel cellular therapies including CAR-T cells have shown promise for small numbers of patients with chemoresistant Richter transformation (174).

In rare cases, CLL may undergo transformation to B-cell prolymphocytic leukemia (B-PLL), which is characterized by the rapid development of B symptoms, marked leukocytosis, and splenomegaly (175, 176). B-PLL was previously defined by the presence of >55% prolymphocytes in the peripheral blood (177). However, the updated World Health Organization classification no longer recognizes B-PLL as a distinct entity; instead, cases of CLL which develop >15% prolymphocytes in the peripheral blood and/or bone marrow are designated as ‘prolymphocytic progression of CLL/SLL’ (178). Although data is sparse, allogeneic HCT may be considered for eligible patients with prolymphocytic progression of CLL due to the poor prognosis of this disease without HCT (179, 180). In one CIBMTR study including 11 patients with B-PLL, 1-year OS was 48% and PFS was 33% after allogeneic HCT (181).

## Discussion

Given the risks of transplant-related morbidity and mortality, the decision to proceed to allogeneic HCT for CLL is not one to be taken lightly. Indeed, the emergence of well-tolerated and effective targeted agents for CLL have relegated allogeneic HCT to later lines of therapy, and it is now commonly considered a treatment of last resort for patients who lack other options. However, allogeneic HCT remains an important part of the therapeutic armamentarium for CLL. It is perhaps the only treatment able to alter the natural history of the disease through the GVL effect, which may delay CLL progression and potentially result in cure. With the ability to achieve long-lasting remissions in >30-50% of patients, allogeneic HCT is still a valuable option for patients with heavily pretreated CLL, high-risk genetic features, and resistance to BTK and/or BCL2 inhibitors. In addition, advances in transplantation technique have increased the availability of this therapy to older age groups while still reducing TRM risks to <10-15% in contemporary studies.

While the next generation of CLL treatments such as the non-covalent BTK inhibitor pirtobrutinib have demonstrated encouraging response rates, its median PFS of 18 months among patients previously treated with both BTK and BCL2 inhibitors indicates that additional treatment options will still be needed for CLL, including allogeneic HCT in appropriate candidates (182). CAR-T cell and CAR-NK cell therapies are also in development for CLL, but none have received regulatory approval for this indication and further work is needed to overcome the intrinsic T-cell dysfunction associated with CLL (161, 183, 184). Allogeneic HCT thus remains the best-established cellular therapy for CLL at the present time. Further investigation is needed to determine if the efficacy of allogeneic HCT can be further improved by the addition of novel targeted, immune, and/or cellular therapies as consolidation or maintenance after transplantation. Given that patients with CLL remain at particularly high risk of GVHD, studies on GVHD prophylaxis specifically in this population are needed to confirm if ATG-Thymoglobulin®, PTCy, or other agents may confer protection against GVHD without compromising GVL.

Patients with CLL remain at particularly high risk of GVHD, studies on GVHD prophylaxis specifically in this population are needed to confirm if ATG-Thymoglobulin^®^, PTCy, or other agents may confer protection against GVHD without compromising GVL. Finally, ongoing research is required to re-evaluate the risks and benefits of allogeneic HCT vis-à-vis the growing number of emerging therapeutic options for multiply relapsed CLL.

In addition to the recent advances in HCT outlined in this article, it is anticipated that further improvements in bridging therapy, donor selection, GVHD prophylaxis, RIC protocols, and supportive care are likely to make allogeneic HCT increasingly safe and effective for the small but deserving group of patients with CLL who are likely to benefit from this procedure in the future.

## Author contributions

All authors listed have made a substantial, direct, and intellectual contribution to the work and approved it for publication.
